# Health Care Utilization Among Texas Veterans Health Administration Enrollees Before and After Hurricane Harvey, 2016-2018

**DOI:** 10.1001/jamanetworkopen.2021.38535

**Published:** 2021-12-10

**Authors:** Margaret Carrel, Gosia S. Clore, Seungwon Kim, Mary Vaughan Sarrazin, Eric Tate, Eli N. Perencevich, Michihiko Goto

**Affiliations:** 1 Department of Geographical and Sustainability Sciences, University of Iowa, Iowa City; 2 Department of Internal Medicine, University of Iowa, Iowa City; 3 Center for Access and Delivery Research and Evaluation, Iowa City Veterans Affairs Health Care System, Iowa City, Iowa

## Abstract

**Importance:**

Hurricanes and flooding can interrupt health care utilization. Understanding the magnitude and duration of interruptions, as well as how they vary according to hazard exposure, race, and income, are important for identifying populations in need of greater retention in care.

**Objective:**

To determine how the differential exposure to Hurricane Harvey in August 2017 is associated with changes in utilization of Veterans Health Administration health care.

**Design, Setting, and Participants:**

This is a retrospective cohort analysis of primary care practitioner (PCP) visits, emergency department visits, and inpatient admissions in the Veterans Health Administration among Texas veterans residing in counties impacted by Hurricane Harvey from 2016 to 2018. Data analysis was performed from September 2020 to May 2021.

**Exposures:**

Residential flooding after Hurricane Harvey.

**Main Outcomes and Measures:**

Interrupted time series analysis measured changes in health care utilization over time, stratified by residential flood exposure, race, and income.

**Results:**

Of the 99 858 patients in the cohort, 89 931 (90.06%) were male, and their median (range) age was 58 (21 to 102) years. Compared with veterans in nonflooded areas, veterans living in flooded areas were more likely to be Black (24 715 veterans [33.80%] vs 4237 veterans [15.85%]) and low-income (14 895 veterans [20.37%] vs 4853 veterans [18.15%]). Rates of PCP visits decreased by 49.78% (95% CI, −64.52% to −35.15%) for veterans in flooded areas and by 45.89% (95% CI, −61.93% to −29.91%) for veterans in nonflooded areas and did not rebound until more than 8 weeks after the hurricane. Rates of PCP visits in flooded areas remained lower than expected for 11 weeks among White veterans (−6.99%; 95% CI, −14.36% to 0.81%) and for 13 weeks among racial minority veterans (−7.22%; 95% CI, −14.11% to 0.30%). Low-income veterans, regardless of flood status, experienced greater suppression of PCP visits in the 8 weeks following the hurricane (−13.72%; 95% CI, −20.51% to −6.68%) compared with their wealthier counterparts (−9.63%; 95% CI, −16.74% to −2.26%).

**Conclusions and Relevance:**

These findings suggest that flood disasters such as Hurricane Harvey may be associated with declines in health care utilization that differ according to flood status, race, and income strata. Patients most exposed to the disaster also had the greatest delay or nonreceipt of care.

## Key Points

**Question:**

Was residential flooding due to Hurricane Harvey in August 2017 associated with health care utilization among veterans in Texas?

**Findings:**

This cohort study including 99 858 patients found greater and longer lasting declines in primary care visits, emergency department visits, and inpatient admissions among veterans with flooded residences, racial minority veterans, and low-income veterans.

**Meaning:**

These findings suggest that veteran patients most exposed to Hurricane Harvey also had the greatest delay in or nonreceipt of health care.

## Teaser

This cohort study examines how the differential exposure to Hurricane Harvey in August 2017 is associated with changes in utilization of Veterans Health Administration (VHA) health care.

## Introduction

In late August 2017, a 4-day rain event triggered by Hurricane Harvey deposited more than 270 trillion gallons of water across southeastern Texas, with some areas receiving more than 50 inches of precipitation. In the Houston metropolitan area, one-third of the land was underwater and more than 100 000 homes were destroyed. Floodwaters pose risk in multiple ways over both short and long time spans, including direct exposure to contaminants (eg, petrochemicals and bacteria), injuries from flood-borne debris, blocked access to health care facilities, and the growth of mold in waterlogged walls and floors.^1,2,3,4^ A growing body of research indicates that exposure to hurricanes and flooding has wide-ranging health impacts in the US.^5,6,7,8,9,10,11,12,13,14^ Utilization of health care also changes, with patients delaying, cutting back on, or terminating treatments; facing greater barriers to care; or shifting care to telehealth visits or to facilities that are not disaster affected.^13,15,16,17,18,19^

Changes in health status and health care utilization following disasters are not universal in populations exposed to hurricanes or flooding, however. The presence, magnitude, and duration of negative outcomes following disasters are often highly conditioned on race and ethnicity and socioeconomic status. Indeed, differential social vulnerabilities before flooding or other disaster exposures frequently exacerbate and accelerate differential trajectories following disasters.^15,20,21^ Differences in exposure to flooding after hurricanes also break down along race and ethnicity and socioeconomic status: low-income and racial and ethnic minority populations often experience more flooding than do White and wealthy populations, as has been observed following Hurricanes Katrina, Sandy, and Harvey, because of historical and current structural inequities.^22,23,24,25,26,27^

Prior research into health outcomes associated with hurricanes relied on surveys and self-reported outcomes, focused only on short-term or long-term changes in outcomes rather than tracking consistently over time, poorly characterized race and income dimensions, had assessment of hurricane exposure that was nonspecific (eg, at the county scale), or focused on facilities rather than home residence. The current study uses 3 years of electronic health records, coupled with addresses of patients and a high-resolution flood map, to assess how utilization of Veterans Health Administration (VHA) services changed in the Harvey-impacted area. We investigate immediate and long-term changes in visits to primary care practitioner (PCPs; eg, physicians, nurse practitioners, and physician assistants who provide primary care), emergency department (ED) visits, and inpatient admissions, stratified by the flood status of veteran residences, in the weeks and months following hurricane-associated flooding. We further examine how posthurricane changes to health care utilization vary according to the race and income of veterans.

## Methods

 This cohort study was approved by the institutional review board at the University of Iowa with a waiver of informed consent. Consent was waived because the study is a retrospective, secondary data analysis of health records with no direct contact with patients, in accordance with 45 CFR §46. This study follows the Strengthening the Reporting of Observational Studies in Epidemiology (STROBE) reporting guidelines for cohort studies.

Data were obtained from the VA Corporate Data Warehouse. A cohort of veterans was created according to residence in the 41 counties declared eligible for individual assistance by Federal Emergency Management Administration in August 2017 and active use of the VHA that year. Veterans were excluded from the cohort if their residential address was geocoded only to the zip code level or coarser or if they resided in group quarters (2549 veterans).

A flood depth grid developed by the Federal Emergency Management Administration with a 3- by 3-m horizontal resolution was used to determine the residential flood status of veterans in the cohort.^28^ The latitude and longitude of residential location of veterans, assigned by the VHA, was overlaid on the flood depth raster from Federal Emergency Management Administration to assign residential flood status (eFigure 1 in the Supplement). χ^2^ tests indicated differences in veteran characteristics according to residential flood status during Hurricane Harvey. Veteran characteristics included demographic characteristics (age, sex, and self-reported race), VHA priority level, and comorbidities. Race was assessed because of prior research indicating racial and ethnic variations in both health care utilization and disaster exposure.^22,23,24,25,26,27^ VHA priority is assigned on the basis of military service, disability rating, income level, and other factors. Veterans are categorized as low income if they do not have a service-related disability and have income below a VA-determined adjusted income limit (based on resident zip code) or are eligible for Medicaid. Comorbidities included 14 medical conditions defined by the Charlson Comorbidity Index.^29^

Weekly number of visits to a PCP, visits to a VHA ED, and inpatient admissions to a VHA facility during 2016, 2017, and 2018 were assessed. Healthcare utilization was calculated per 100 000 veterans for each of the 156 weeks in the study period. Interrupted time series (ITS) analysis was used to explore whether changes in PCP visits, ED visits, and inpatient admissions following Hurricane Harvey varied according to veteran residential flood status.

### Statistical Analysis

Segmented linear regression models with autoregressive errors were fit (eAppendix in the Supplement). In addition to the baseline trend that existed before the hurricane, 3 parameters to estimate the changes associated with the timing of the hurricane were included in the models: (1) a dummy variable to indicate the posthurricane status, interpreted as the immediate change at the time of the hurricane; (2) the number of weeks after the hurricane to indicate linear changes in trends; and (3) the number of weeks after the hurricane with logarithmic transformation indicating decaying associations. Autoregressive terms were included in models to account for seasonality. The number of autoregressive parameters was determined using a stepwise method that sequentially removed autoregressive parameters until those remaining were significant (*P* < .05).

All ITS models examined the 86 weeks before and 70 weeks after the event. Prehurricane trend (weeks between January 1, 2016, and August 20, 2017), the change in level of the visit rate (week of August 20, 2017), and the change in trend after the hurricane (weeks between August 27, 2017, and December 31, 2018) compared with before the hurricane were estimated. To estimate changes in utilization following the hurricane, estimates were calculated from 2 models derived from a fitted segmented autoregressive linear model. Point estimates from a model with all parameters included (actual model) and from a model only with baseline trend and autoregressive terms (counterfactual model) were calculated. Absolute change was estimated by subtracting a point estimate from the actual model (estimated actual value) from an estimate from the counterfactual model (expected value without hurricane), and a relative change was calculated by dividing an estimate from the actual model by an estimate from the counterfactual model. Bootstrapping methods using ITS parameter estimates were used to generate estimates and 95% CIs of the absolute and relative changes in health care visits immediately and in the weeks after the hurricane.^30^ The 95% CIs were also used to determine at what week health care visits returned to expected levels. Secondary ITS analysis using stratification and interaction terms examined the associations between hurricane-related flooding and health care for White vs racial minority subgroups and low income vs non–low-income subgroups.

Statistical analysis was completed in SAS statistical software version 9.4 (SAS Institute). Spatial analysis was completed in ArcMap software version 10.7.1 (Esri). Data analysis was performed from September 2020 to May 2021.

## Results

A total of 99 858 veterans were included in the analytical cohort. The majority (89 931 veterans [90.06%]) were male, and the median (range) age was 58 (18-102) years (Table 1). Of these, 73 120 veterans (73.2%) were classified as having residential flood exposure. Significant differences were observed between the composition of veterans with residential flood exposure vs those whose addresses were not flooded. Veterans residing in flooded areas were more likely than veterans without residential flood exposure to be female (7481 veterans [10.23%] vs 2466 veterans [9.15%]) and were slightly younger (mean [SD] age, 58.1 [16.9] vs 59.4 [16.7] years). The cohort living in flooded areas had a higher proportion of Black veterans (24 715 veterans [33.80%] vs 4237 veterans [15.85%]) and a higher proportion of veterans in the low-income priority category 5 (14 895 veterans [20.37%] vs 4853 veterans [18.15%]). Veterans in flooded areas also had higher proportions of most Charlson comorbidities. The majority of veterans in the study cohort resided in Harris County, where the city of Houston is located (eFigure 1 in the Supplement).

**Table 1.** Characteristics of Study Cohort

Characteristic	Participants, No. (%)^a^	
	Without residential flooding (n = 26 738)	With residential flooding (n = 73 120)
Sex		
Female	2446 (9.15)	7481 (10.23)
Male	24 292 (90.85)	65 639 (89.77)
Age, y		
Mean (SD)	59.4 (16.7)	58.1 (16.9)
Median (range)	64 (21-102)	61 (18-102)
18-39	4834 (18.08)	13 834 (18.92)
40-64	8892 (33.26)	27 593 (37.74)
65-79	10 462 (39.13)	25 697 (35.14)
≥80	2550 (9.54)	5996 (8.20)
Race		
Black	4237 (15.85)	24 715 (33.80)
Multiracial	308 (1.15)	871 (1.19)
White	20 448 (76.48)	43 150 (59.01)
Other	522 (1.95)	1544 (2.11)
Missing	1223 (4.57)	2840 (3.88)
Charlson Comorbidity Index		
Diabetes	8251 (30.86)	23 399 (32.00)
Chronic obstructive pulmonary disease	4807 (17.98)	12 246 (16.75)
Diabetes with complications	3702 (13.85)	11 356 (15.53)
Kidney disease	3074 (11.50)	9410 (12.87)
Peripheral vascular disease	2702 (10.11)	7899 (10.80)
Cerebrovascular disease	2430 (9.09)	7087 (9.69)
Malignant tumor	2416 (9.04)	6909 (9.45)
Congestive heart failure	2262 (8.46)	6890 (9.42)
Mild liver disease	1930 (7.22)	6822 (9.33)
Dementia	1128 (4.22)	3636 (4.97)
Myocardial infarction	637 (2.38)	2076 (2.84)
Rheumatic disease	529 (1.98)	1366 (1.87)
Metastatic solid tumor	347 (1.30)	1247 (1.71)
Peptic ulcer disease	278 (1.04)	879 (1.20)
Hemiplegia or paraplegia	230 (0.86)	875 (1.20)
AIDS	155 (0.58)	774 (1.06)
Moderate or severe liver disease	182 (0.68)	647 (0.88)
Priority category		
1 (disability >50%, Medal of Honor)	10 834 (40.52)	28 017 (38.32)
2-4 (disability <50%, prisoner of war, Purple Heart, catastrophically disabled)	6248 (23.37)	17 373 (23.75)
5 (low income)	4853 (18.15)	14 895 (20.37)
6-8 (other)	4803 (17.96)	12 835 (17.55)

^a^ *P* < .001 for all characteristics for veterans with residential flooding vs those without residential flooding.

**Figure 1.**
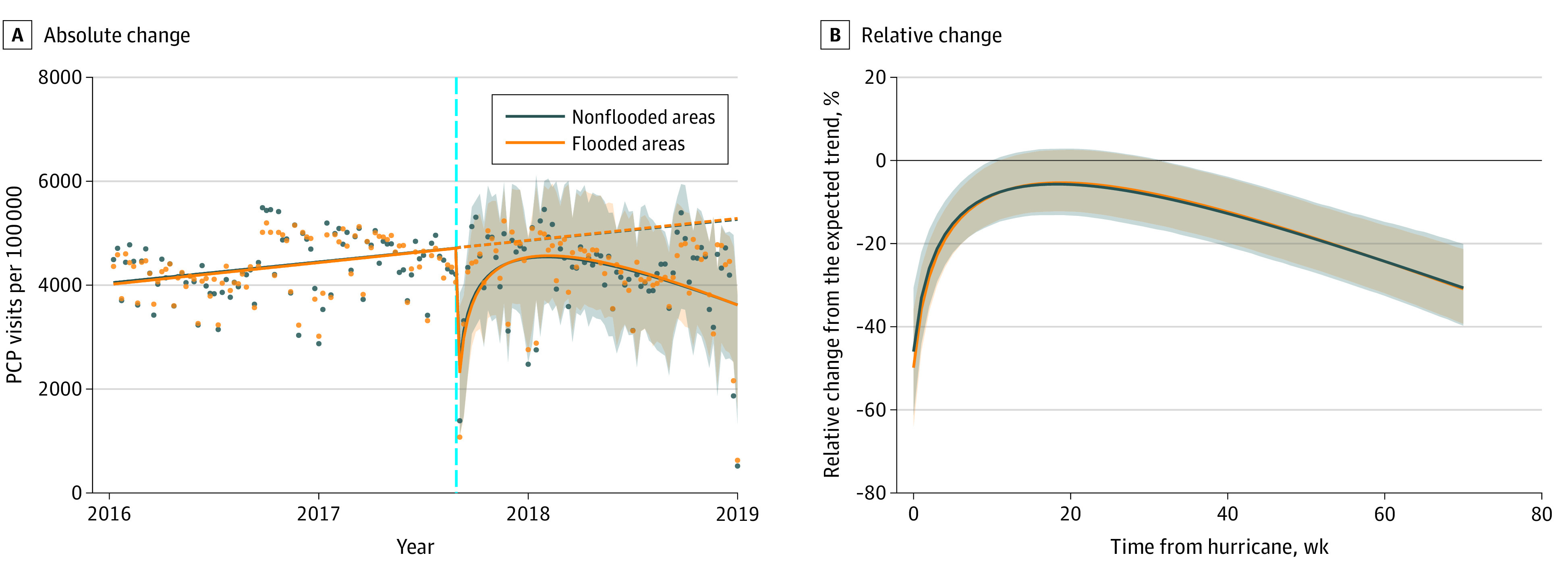
Absolute and Relative Changes in Primary Care Practitioner (PCP) Visits Among Veterans With Flooded and Nonflooded Residences A, Solid lines fit observed weekly data points (dots), dashed line indicates trends if no hurricane had occurred, vertical cyan line denotes date of hurricane, and shaded areas represent 95% CIs. B, Solid lines denote weekly percentage relative change from expected trend, and shaded areas represent 95% CIs.

The ITS analysis indicated that veterans in flooded and nonflooded areas had similar rates of PCP visits over time and comparable changes following Hurricane Harvey (Figure 1). Veterans in flooded areas had 2345.55 fewer PCP visits (95% CI, −3100.75 to −1628.16 visits) the week Hurricane Harvey made landfall, a 49.78% (95% CI, −64.52% to −35.15%) decrease (Table 2). Veterans outside of flooded areas had 2163.82 fewer PCP visits (95% CI, −2983.48 to −1385.21 visits) that week, a 45.89% (95% CI, −61.93% to −29.91%) decrease. Veterans experiencing flooding had larger absolute and relative decreases in PCP visits in weeks 1, 2, 4, and 8 following the hurricane. PCP visits for veterans, regardless of flood exposure, were not significantly different than the prehurricane period by 3 months after the hurricane (week 12 for veterans with and week 11 for veterans without residential flooding).

**Table 2.** Absolute and Relative Effects of Hurricane Harvey on PCP Visits, ED Visits, and Inpatient Admissions in Veterans With Flooded and Nonflooded Residential Status at Weeks 0, 1, 2, 4, and 8 and, if Applicable, the Week When Changes Were Not Significantly Different Than Before the Hurricane

Visit type and week	With residential flooding		Without residential flooding	
	Absolute decline, visits per 100 000 (95% CI)	Relative decline, % (95% CI)	Absolute decline, visits per 100 000 (95% CI)	Relative decline, % (95% CI)
PCP visit				
0	−2345.55 (−3100.75 to −1628.16)	−49.78 (−64.52 to −35.15)	−2163.82 (−2983.48 to −1385.21)	−45.89 (−61.93 to −29.91)
1	−1685.50 (−2218.15 to −1148.52)	−35.69 (−46.46 to −24.89)	−1559.73 (−2137.84 to −976.92)	−33.00 (−44.67 to −21.27)
2	−1321.20 (−1778.00 to −874.64)	−27.91 (−36.71 to −19.21)	−1226.99 (−1722.77 to −742.32)	−25.89 (−35.43 to −16.38)
4	−901.11 (−1275.10 to −517.74)	−18.95 (−26.09 to −11.4)	−844.57 (−1250.47 to −428.46)	−17.73 (−25.50 to −9.49)
8	−506.96 (−871.36 to −150.71)	−10.54 (−17.49 to −3.36)	−488.96 (−884.45 to −102.31)	−10.15 (−17.69 to −2.3)
12	−339.04 (−708.85 to 27.04)	−6.97 (−13.98 to 0.59)	−366.71 (−773.04 to 29.64)^a^	−7.54 (−15.23 to 0.65)^a^
ED visit				
0	−204.65 (−317.56 to −95.04)	−20.87 (−32.03 to −9.96)	−127.57 (−229.42 to −28.7)	−20.98 (−37.08 to −4.92)
1	−180.12 (−260.79 to −98.65)	−18.31 (−26.34 to −10.22)	−100.54 (−173.31 to −27.05)	−16.48 (−28.19 to −4.64)
2	−168.01 (−237.37 to −100.97)	−17.02 (−23.54 to −10.47)	−86.36 (−148.93 to −25.89)	−14.11 (−23.66 to −4.37)
4	−156.72 (−213.01 to −98.86)	−15.78 (−20.95 to −10.28)	−71.38 (−122.16 to −19.18)	−11.60 (−19.29 to −3.30)
8	−152.86 (−207.36 to −99.12)	−15.21 (−20.11 to −10.28)	−60.77 (−109.94 to −12.29)	−9.77 (−17.13 to −2.06)
Inpatient visit				
0	−64.47 (−114.74 to −16.71)	−19.59 (−34.07 to −5.22)	−49.43 (−105.41 to 3.74)	−21.49 (−44.38 to 1.70)
1	−46.38 (−81.83 to −10.63)	−14.03 (−24.61 to −3.34)	−30.55 (−70.03 to 9.26)	−13.20 (−30.04 to 4.22)
2	−36.72 (−81.83 to −10.63)	−11.06 (−19.84 to −2.21)	−20.11 (−53.97 to 12.99)	−8.62 (−22.67 to 5.87)
4	−26.19 (−51.08 to −0.67)	−7.82 (−14.81 to −0.21)	−8.05 (−35.77 to 20.36)	−3.33 (−14.67 to 9.46)
8	−17.84 (−42.11 to 5.86)	−5.24 (−11.98 to 1.84)	3.32 (−23.68 to 29.73)	1.65 (−9.54 to 13.86)

Abbreviations: ED, emergency department; PCP, primary care practitioner.

^a^ These data are for week 11, not week 12.

**Figure 2.**
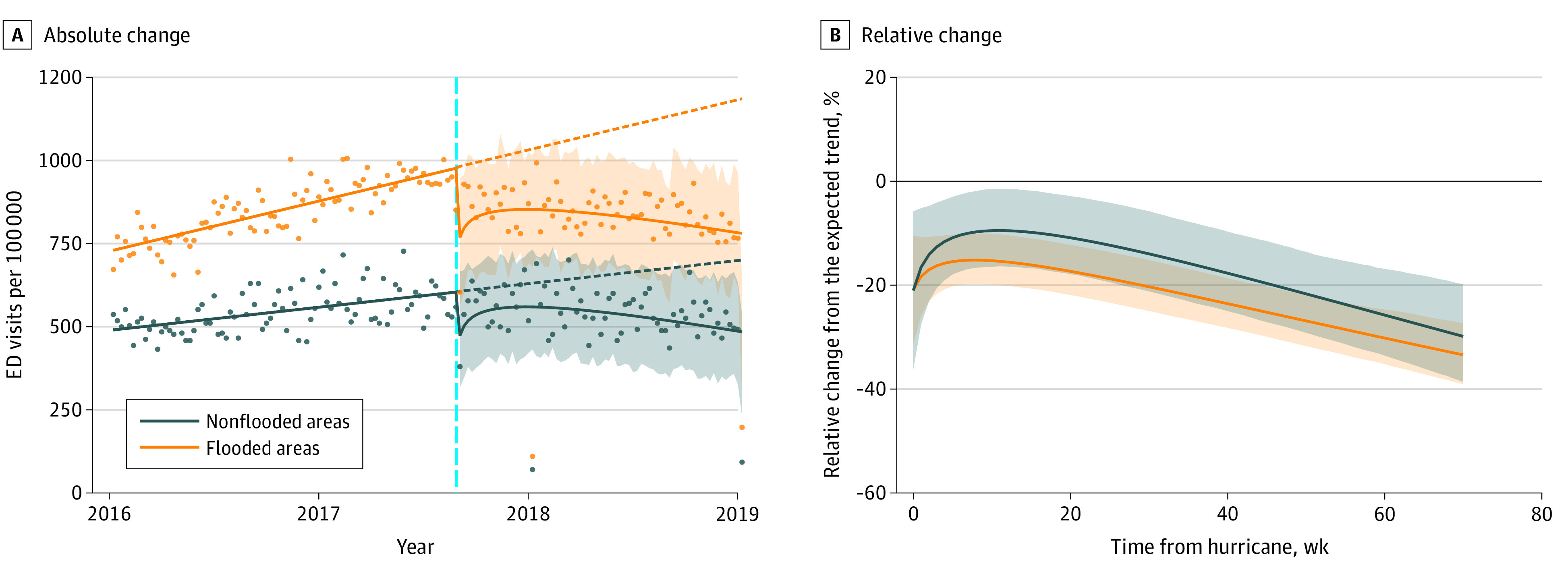
Absolute and Relative Changes in Emergency Department (ED) Visits Among Veterans With Flooded and Nonflooded Residences A, Solid lines fit observed weekly data points (dots), dashed lines indicate trends if no hurricane had occurred, vertical cyan line denotes date of hurricane, and shaded areas represent 95% CIs. B, Solid lines denote weekly percentage relative change from expected trend, and shaded areas represent 95% CIs.

**Figure 3.**
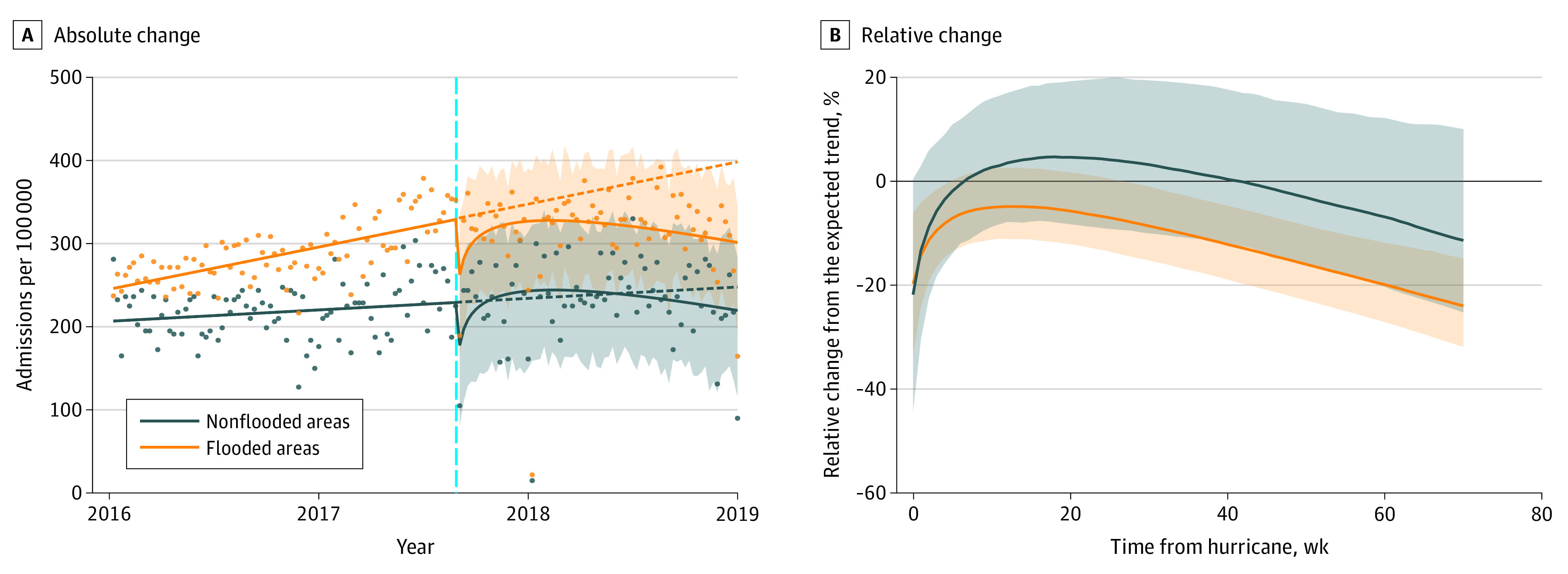
Absolute and Relative Changes in Inpatient Admissions Among Veterans With Flooded and Nonflooded Residences A, Solid lines fit observed weekly data points (dots), dashed lines indicate trends if no hurricane had occurred, vertical cyan line denotes date of hurricane, and shaded areas represent 95% CIs. B, Solid lines denote weekly percentage relative change from expected trend, and shaded areas represent 95% CIs.

Veterans with flooded residences had consistently higher rates of ED visits and inpatient admissions than veterans without residential flooding over the years of the study (Figure 2 and Figure 3). ED visits among veterans with flooded housing decreased by 20.87% (95% CI, −32.03% to −9.96%) the week of the hurricane, whereas those among veterans without flooded housing decreased by 20.98% (95% CI, −37.08% to −4.92%) (Table 2). ED visits among all veterans remained significantly lower than expected during the 70 weeks after Hurricane Harvey, regardless of flood status. Inpatient admissions among veterans with flooded residences significantly decreased in the month following the hurricane and did not return to prestorm levels until after 4 weeks. In contrast, veterans without flooded residences exhibited no significant changes in inpatient admission rates.

Although both the residentially flooded and nonflooded veteran cohorts exhibited significant decreases in PCP visits immediately following Hurricane Harvey, there were differences in the absolute and relative decline when veterans were stratified by race in exploratory subgroup analysis. Rates of PCP visits were similar across veterans with and without residential flooding when categorized by race (eFigure 2 in the Supplement). In the 2 months following the hurricane, racial minority veterans with residential flooding had higher absolute and relative declines in PCP visits than White veterans who experienced residential flooding (eTable 1 in the Supplement). White veterans, regardless of flood status, had significantly lower than expected decreases in PCP visits until week 11 following Hurricane Harvey (−6.99%; 95% CI, −14.36% to 0.81%). By contrast, veterans who were not White and who experienced residential flooding remained at lower-than-expected rates for longer, until week 13 (−7.22%; 95% CI, −14.11% to 0.30%). Overall, however, there were no significantly different patterns of PCP visits in the posthurricane period for White vs racial minority patients (eTable 2 in the Supplement). For veterans without residential flooding, PCP rates had similar responses to the hurricane: PCP visits among racial minority veterans without flood exposure remained significantly lower than expected until week 10 (−7.80%; 95% CI, −15.66% to 0.66%), whereas PCP visits among White veterans without flood exposure remained low until week 11 (−7.59%; 95% CI, −15.45% to 0.8%).

Over the duration of the study, the highest rates of ED visits were observed in racial minority veterans who lived in the area flooded by Hurricane Harvey (eFigure 3 in the Supplement). Rates of ED visits were also higher in racial minority veterans who lived in nonflooded areas than White veterans living in nonflooded areas. These differences before the hurricane were significant (eTable 2 in the Supplement). Rates of ED visits among White veterans without flood exposure did not change significantly after the hurricane, with no period with a lower number of visits than expected (week 0, −16.76%; 95% CI, −34.26% to 0.82%) (eTable 1 in the Supplement). By contrast, rates of ED visits for White veterans with flood exposure and racial minority veterans in both flooded and nonflooded areas remained significantly lower than expected, even by 70 weeks after the hurricane.

Similar to ED visits, inpatient admission rates were highest among racial minority veterans who lived in flooded areas during the years of the study (eFigure 4 in the Supplement). Inpatient admission rates did not change significantly for White veterans without residential flood exposure and were significantly lower only at weeks 0 (−39.68%; 95% CI, −77.03% to −0.65%) and 1 (−29.3%; 95% CI, −56.97% to −0.52%) for racial minority veterans without flood exposure (eTable 1 in the Supplement). White veterans with flood exposure had inpatient admission rates return to expected levels at week 8, whereas racial minority veterans in flooded areas had inpatient admission rates remain significantly lower than expected for only 1 week after the hurricane. No significant differences in admissions between White and racial minority veterans were observed (eTable 2 in the Supplement).

When veterans were stratified by low-income priority status for exploratory subgroup analysis, steeper increases in PCP visit rates before Hurricane Harvey were observed in low-income veterans across flood status (eFigure 5 in the Supplement). PCP visit rates decreased by 56.4% (95% CI, −71.17% to −41.97%) among low-income veterans with flood exposure in week 0 and by 44.39% (95% CI, −60.21% to −28.73%) among low-income veterans without flood exposure. PCP visit rates remained significantly lower than expected at least until the end of the study period for low-income veterans vs higher income veterans (−13.72% [95% CI, −20.51% to −6.68%] vs −9.63% [95% CI, −16.74% to −2.26%]), regardless of flood status, following Hurricane Harvey (eTable 3 in the Supplement). Among non–low-income veterans, PCP visits rebounded by week 10 for veterans in flooded areas and week 9 for veterans without flood exposure.

Rates of ED visits among low-income veterans were higher, across flood status, than for their non–low-income counterparts during the study period (eFigure 6 in the Supplement). These differences were significant in the weeks before the hurricane (eTable 4 in the Supplement). Across priority categories, veterans with residential flood exposure experienced decreases in ED visits that extended to the end of the study period, whereas non–low-income veterans without flood exposure had rates of ED visits that rebounded by week 8 (eTable 3 in the Supplement). Low-income veterans living in nonflooded areas experienced no decreases in ED visits until week 2.

Low-income veterans with residences that flooded during Hurricane Harvey had higher rates of inpatient admissions than other veterans during the study period (eFigure 7 in the Supplement). Although inpatient admission rates did not change for non–low-income veterans without flooded residences and returned to baseline for non–low-income veterans with flooded residences by week 3, inpatient admission rates for low-income veterans in both flooded and nonflooded areas remained significantly lower than expected until week 5 (eTable 3 in the Supplement). Low-income veterans in flooded areas had significantly lower inpatient admissions following the hurricane than did their non–low-income counterparts (eTable 4 in the Supplement).

## Discussion

Hurricane Harvey–impacted counties of Texas were home to nearly 100 000 active users of the VHA at the time of the storm, mostly in Harris County. In this cohort study, when veterans’ residences were categorized as being in a flooded area vs not, higher percentages of Black and low-income veterans were observed in flooded vs nonflooded areas. This is consistent with prior research on populations exposed to Hurricane Harvey flooding.^23,24,25^

Hurricane Harvey’s landfall was associated with immediate and significant declines in PCP visits for both veterans with and without flooded residences, with the rate of visits decreasing by nearly half. These declines persisted for over 2 months, remaining significantly lower than expected rates beyond 8 weeks. Rates of ED visits among veterans living in the Hurricane Harvey–impacted counties never returned to their expected rates from prehurricane baseline, whereas inpatient admissions were not strongly changed in the weeks following the hurricane.

Persistently higher rates of ED visits and inpatient admissions were observed among veterans living in areas flooded by Hurricane Harvey over the 3-year period of the study. Even in the years before and after Hurricane Harvey, veterans who resided in areas that were subject to flooding in late 2017 had higher rates of use of the Houston VA medical center, visiting the ED, or being admitted for procedures. This is consistent with higher levels of comorbidities in the flooded cohort.

Differences in health care utilization by race and income were observed over the 3-year study period. The higher rates of ED visits in veterans with residential flood exposure vs those without were associated primarily with higher rates of ED visits for racial minority veterans, as were the higher rates of inpatient admissions. Low-income veterans also had higher rates of ED visits and inpatient admissions than other veterans. Higher rates of ED use in the VHA have been linked to lower income and greater comorbidity.^31,32^

Although rates of PCP visits were similar across flood status and race, racial minority veterans who experienced residential flooding took 2 more weeks to rebound than did their White counterparts. Inpatient admission rates among low-income veterans, in both flooded and nonflooded areas, remained significantly lower than expected for 5 weeks, whereas non–priority category 5 veterans either experienced no declines (nonflooded residence) or a return to baseline after 3 weeks (flooded residence). Delays in inpatient admissions can result in greater adverse outcomes for conditions such as cancer and cardiovascular disease.^33^

The study did not limit health care utilization among veterans in the cohort to only those VHA locations within the study area. VHA data capture all health care visits in VHA facilities, so the declines in care that were observed were not the result of veterans seeking care in other areas of Texas or in other states. Veterans can be seen at any VHA facility, even if they flee the area affected by a hurricane, suggesting that the declines in utilization observed in the VHA data may be smaller than declines in other health care systems where patients are limited in the facilities they can use. The size of the VHA and its integrated systems support means that it can often better respond to interruptions in care than smaller service providers.^34^

Both immediate and long-term changes in health care utilization can occur because of exposure to hurricanes, floods, and other natural disasters. Examining both short-term and long-term changes in health care use is important, given that consequences of exposure to disasters or disruption in health care play out at different timescales. Understanding varying responses in health care utilization to disaster events is also critical, as weather events such as Hurricane Harvey are expected to increase in frequency and intensity.^35^ Although a shift to telehealth provision of care can alleviate some of the disruptions in care caused by physical barriers in access, it cannot replace in-person provision of services such as immunizations. Telehealth is also not an immediately viable replacement for care when disasters disrupt electrical grids and cellular telephone networks. The VHA has made use of mobile health units during the aftermath of hurricanes and other disasters, and their wider usage, as well as targeted, active follow-up by VHA with patients who have not maintained regular PCP visits, could alleviate some of the declines observed in this study and are options available to health care practitioners outside the VHA.^36,37^

### Limitations

Flexibility in seeking care with the VHA is a limitation of the study, given that many veterans have dual eligibility for private sector care. The study is unable to capture their PCP visits, inpatient admission, or ED visits outside of VHA facilities. An additional limitation is that veterans without residential flooding could still face barriers, such as flooded roads, to health care utilization that are not captured in this study. The study also does not consider what conditions were linked to the PCP and ED visits and inpatient admissions during the study period and so cannot address changes in health status associated with hurricane exposure, such as emergent conditions or worsening of health indicators. In addition, low-income veterans can be assigned priority categories other than 5 if they have service-related disabilities, so we may not be fully capturing the experience of low-income veterans.

## Conclusions

In conclusion, this cohort study highlights the disparities in both flood exposure status and health care utilization by race and income prior to Hurricane Harvey associated with structural inequities that are exacerbated by disasters. Reducing these inequities in times between disasters is crucial to mitigating unmet health needs in times of crisis. Understanding how outpatient PCP visits, ED visits, and inpatient admissions change following extensive and catastrophic flooding, in the short and long term, vary across social groups is important for targeting greater retention in patients most at-risk of both disaster exposure and delay or nonreceipt of care.

## Notes

Supplement. **eTable 1.** Absolute and Relative Effects of Hurricane Harvey on PCP Visits, ED Visits and Inpatient Admissions in White and Non-White Veterans With Flooded and Nonflooded Residential Status at Weeks 0, 1, 2, 4, 8 and, if Applicable, the Week That Visits Were No Longer Significantly Different Than Expected **eTable 2.** Estimates and *P* Values for ITS Models for Healthcare Utilization Stratified by Flood Status With Interaction Terms for Race **eTable 3.** Absolute and Relative Effects of Hurricane Harvey on PCP Visits, ED Visits and Inpatient Admissions in Priority Category 5 and Non–Priority Category 5 Veterans With Flooded and Nonflooded Residential Status at Weeks 0, 1, 2, 4, 8 and, if Applicable, the Week That Visits Were No Longer Significantly Different Than Expected **eTable 4.** Estimates and *P* Values for ITS Models for Healthcare Utilization Stratified by Flood Status With Interaction Terms for Priority Category (Income) **eFigure 1.** Flooded Areas in Texas Following Hurricane Harvey With Locations of VHA Care (Houston VA Medical Center [VAMC] and Community Based Outpatient Clinics [CBOCs]) and Total Veterans in Disaster Declared Counties **eFigure 2.** Absolute and Relative Changes in PCP Visits Among Veterans With Flooded (Red) and Nonflooded (Blue) Residences Who Were White (A, B) and Non-White (C,D) **eFigure 3.** Absolute and Relative Changes in ED Visits Among Veterans With Flooded (Red) and Nonflooded (Blue) Residences Who Were White (A, B) and Non-White (C,D) **eFigure 4.** Absolute and Relative Changes in Inpatient Admissions Among Veterans With Flooded (Red) and Nonflooded (Blue) Residences Who Were White (A, B) and Non-White (C,D) **eFigure 5.** Absolute and Relative Changes in PCP Visits Among Veterans With Flooded (Red) and Nonflooded (Blue) Residences Who Were Priority Category 5 (A, B) and Non–Priority Category 5 (C,D) **eFigure 6.** Absolute and Relative Changes in ED Visits Among Veterans With Flooded (Red) and Nonflooded (Blue) Residences Who Were Priority Category 5 (A, B) and Non–Priority Category 5 (C,D) **eFigure 7.** Absolute and Relative Changes in Inpatient Admissions Among Veterans With Flooded (Red) and Nonflooded (Blue) Residences Who Were Priority Category 5 (A, B) and Non–Priority Category 5 (C,D) **eAppendix.** Example SAS Code for ITS Models and Bootstrapping of Confidence Intervals
