# Supplementary material for: Health Care Utilization Among Texas Veterans Health Administration Enrollees Before and After Hurricane Harvey, 2016-2018
Source: JAMA Netw Open. 2021 Dec 10;4(12):e2138535. doi: 10.1001/jamanetworkopen.2021.38535 (PMC8665372; doi:10.1001/jamanetworkopen.2021.38535)
Supplement: Supplement. — eTable 1. Absolute and Relative Effects of Hurricane Harvey on PCP Visits, ED Visits and Inpatient Admissions in White and Non-White Veterans With Flooded and Nonflooded Residential Status at Weeks 0, 1, 2, 4, 8 and, if Applicable, the Week That Visits Were No Longer Significantly Different Than Expected eTable 2. Estimates and P Values for ITS Models for Healthcare Utilization Stratified by Flood Status With Interaction Terms for Race eTable 3. Absolute and Relative Effects of Hurricane Harvey on PCP Visits, ED Visits and Inpatient Admissions in Priority Category 5 and Non–Priority Category 5 Veterans With Flooded and Nonflooded Residential Status at Weeks 0, 1, 2, 4, 8 and, if Applicable, the Week That Visits Were No Longer Significantly Different Than Expected eTable 4. Estimates and P Values for ITS Models for Healthcare Utilization Stratified by Flood Status With Interaction Terms for Priority Category (Income) eFigure 1. Flooded Areas in Texas Following Hurricane Harvey With Locations of VHA Care (Houston VA Medical Center [VAMC] and Community Based Outpatient Clinics [CBOCs]) and Total Veterans in Disaster Declared Counties eFigure 2. Absolute and Relative Changes in PCP Visits Among Veterans With Flooded (Red) and Nonflooded (Blue) Residences Who Were White (A, B) and Non-White (C,D) eFigure 3. Absolute and Relative Changes in ED Visits Among Veterans With Flooded (Red) and Nonflooded (Blue) Residences Who Were White (A, B) and Non-White (C,D) eFigure 4. Absolute and Relative Changes in Inpatient Admissions Among Veterans With Flooded (Red) and Nonflooded (Blue) Residences Who Were White (A, B) and Non-White (C,D) eFigure 5. Absolute and Relative Changes in PCP Visits Among Veterans With Flooded (Red) and Nonflooded (Blue) Residences Who Were Priority Category 5 (A, B) and Non–Priority Category 5 (C,D) eFigure 6. Absolute and Relative Changes in ED Visits Among Veterans With Flooded (Red) and Nonflooded (Blue) Residences Who Were Priority Category 5 (A, B) a [file jamanetwopen-e2138535-s001.pdf]

Supplemental Online Content

Carrel M, Clore GS, Kim S, et al. Health care utilization among Texas Veterans Health Administration enrollees before and after Hurricane Harvey, 2016-2018. *JAMA Netw Open*. 2021;4(12):e2138535. doi:10.1001/jamanetworkopen.2021.38535

eTable 1. Absolute and Relative Effects of Hurricane Harvey on PCP Visits, ED Visits and Inpatient Admissions in White and Non-White Veterans With Flooded and Nonflooded Residential Status at Weeks 0, 1, 2, 4, 8 and, if Applicable, the Week That Visits Were No Longer Significantly Different Than Expected

eTable 2. Estimates and *P* Values for ITS Models for Healthcare Utilization Stratified by Flood Status With Interaction Terms for Race

eTable 3. Absolute and Relative Effects of Hurricane Harvey on PCP Visits, ED Visits and Inpatient Admissions in Priority Category 5 and Non–Priority Category 5 Veterans With Flooded and Nonflooded Residential Status at Weeks 0, 1, 2, 4, 8 and, if Applicable, the Week That Visits Were No Longer Significantly Different Than Expected

eTable 4. Estimates and *P* Values for ITS Models for Healthcare Utilization Stratified by Flood Status With Interaction Terms for Priority Category (Income)

eFigure 1. Flooded Areas in Texas Following Hurricane Harvey With Locations of VHA Care (Houston VA Medical Center [VAMC] and Community Based Outpatient Clinics [CBOCs]) and Total Veterans in Disaster Declared Counties

eFigure 2. Absolute and Relative Changes in PCP Visits Among Veterans With Flooded (Red) and Nonflooded (Blue) Residences Who Were White (A, B) and Non-White (C,D)

eFigure 3. Absolute and Relative Changes in ED Visits Among Veterans With Flooded (Red) and Nonflooded (Blue) Residences Who Were White (A, B) and Non-White (C,D)

eFigure 4. Absolute and Relative Changes in Inpatient Admissions Among Veterans With Flooded (Red) and Nonflooded (Blue) Residences Who Were White (A, B) and Non-White (C,D)

eFigure 5. Absolute and Relative Changes in PCP Visits Among Veterans With Flooded (Red) and Nonflooded (Blue) Residences Who Were Priority Category 5 (A, B) and Non–Priority Category 5 (C,D)

eFigure 6. Absolute and Relative Changes in ED Visits Among Veterans With Flooded (Red) and Nonflooded (Blue) Residences Who Were Priority Category 5 (A, B) and Non–Priority Category 5 (C,D)

eFigure 7. Absolute and Relative Changes in Inpatient Admissions Among Veterans With Flooded (Red) and Nonflooded (Blue) Residences Who Were Priority Category 5 (A, B) and Non–Priority Category 5 (C,D)

eAppendix. Example SAS Code for ITS Models and Bootstrapping of Confidence Intervals

This supplemental material has been provided by the authors to give readers additional information about their work.

© 2021 Carrel M et al. *JAMA Network Open.*

eTable 1. Absolute and Relative Effects of Hurricane Harvey on PCP Visits, ED Visits and Inpatient Admissions in White and Non-White Veterans With Flooded and Nonflooded Residential Status at Weeks 0, 1, 2, 4, 8 and, if Applicable, the Week That Visits Were No Longer Significantly Different Than Expected

		**Flooded**				
		**White**			**Non-White**	
		**Absolute Decline (95% CI)**	**Relative Decline (95% CI)**		**Absolute Decline (95% CI)**	**Relative Decline (95% CI)**
PCP	Week 0	-2305.22 (-3089.88, -1559.85)	-48.64% (-63.88%, -33.5%)	Week 0	-2396.67 (-3140.65, -1689.95)	-51.32% (-65.93%, -36.8%)
	Week 1	-1646.47 (-2199.9, -1088.54)	-34.66% (-45.79%, -23.5%)	Week 1	-1736.23 (-2260.96, -1207.23)	-37.09% (-47.81%, -26.36%)
	Week 2	-1283.41 (-1758.02, -819.43)	-26.96% (-36.04%, -17.92%)	Week 2	-1370.98 (-1820.98, -931.06)	-29.22% (-37.95%, -20.61%)
	Week 4	-865.75 (-1254.32, -467.43)	-18.09% (-25.49%, -10.27%)	Week 4	-948.42 (-1316.84, -570.75)	-20.12% (-27.2%, -12.65%)
	Week 8	-476.36 (-854.97, -106.22)	-9.84% (-17.05%, -2.37%)	Week 8	-548.48 (-907.46, -197.53)	-11.51% (-18.37%, -4.43%)
	Week 11	-341.75 (-730.73, 37.68)	-6.99% (-14.36%, 0.81%)	Week 13	-349.10 (-710.87, 13.53)	-7.22% (-14.11%, 0.3%)
ED	Week 0	-173.28 (-270.9, -78.53)	-22.57% (-34.8%, -10.54%)	Week 0	-251.43 (-412.5, -95.08)	-19.51% (-31.65%, -7.62%)
	Week 1	-148.87 (-218.61, -78.43)	-19.33% (-28.18%, -10.4%)	Week 1	-226.05 (-341.12, -109.83)	-17.48% (-26.21%, -8.72%)
	Week 2	-136.51 (-196.48, -78.55)	-17.67% (-24.85%, -10.44%)	Week 2	-213.92 (-312.87, -118.29)	-16.49% (-23.57%, -9.35%)
	Week 4	-124.36 (-173.03, -74.33)	-16% (-21.7%, -9.92%)	Week 4	-203.48 (-283.78, -120.93)	-15.59% (-21.19%, -9.6%)
	Week 8	-118.25 (-165.37, -71.79)	-15.03% (-20.44%, -9.55%)	Week 8	-202.58 (-280.33, -125.92)	-15.33% (-20.63%, -9.97%)
Inpatient	Week 0	-74.67 (-125.1 ,-25.71)	-24.61% (-40.48% ,-8.84%)	Week 0	-76.23 (-149.05 ,-5.53)	-20.86% (-39.96% ,-1.56%)
	Week 1	-51.71 (-87.74 ,-15.32)	-16.97% (-28.55% ,-5.24%)	Week 1	-52.83 (-104.86 ,-0.29)	-14.38% (-28.32% ,-0.08%)
	Week 2	-39.47 (-70.45 ,-9.53)	-12.89% (-22.37% ,-3.22%)	Week 2	-40.16 (-84.91 ,3.07)	-10.87% (-22.27% ,0.84%)
	Week 4	-26.17 (-51.31 ,-0.32)	-8.47% (-16.09% ,-0.11%)	Week 4	-26.01 (-62.32 ,11.31)	-6.95% (-16.13% ,3.26%)
	Week 8	-15.73 (-40.08 ,8.27)	-4.99% (-12.45% ,2.8%)	Week 8	-13.88 (-49.04 ,20.78)	-3.59% (-12.57% ,5.8%)
		**Non-Flooded**				
		**White**			**Non-White**	
		**Absolute Decline (95% CI)**	**Relative Decline (95% CI)**		**Absolute Decline (95% CI)**	**Relative Decline (95% CI)**
PCP	Week 0	-2184.54 (-3029.89, -1381.51)	-45.97% (-62.36%, -29.62%)	Week 0	-2064.67 (-2898.17, -1272.9)	-44.99% (-61.76%, -28.29%)
	Week 1	-1574.86 (-2171.09, -973.78)	-33.06% (-45.01%, -21.06%)	Week 1	-1485.17 (-2073.05, -892.52)	-32.27% (-44.47%, -20%)
	Week 2	-1239.11 (-1750.43, -739.24)	-25.95% (-35.72%, -16.22%)	Week 2	-1165.81 (-1669.96, -672.95)	-25.26% (-35.23%, -15.3%)
	Week 4	-853.36 (-1271.99, -424.21)	-17.78% (-25.73%, -9.34%)	Week 4	-798.44 (-1211.2, -375.3)	-17.21% (-25.32%, -8.56%)

© 2021 Carrel M et al. *JAMA Network Open.*

	Week 8	-494.98 (-902.88, -96.21)	-10.2% (-17.91%, -2.15%)	Week 8	-456.02 (-858.19, -62.83)	-9.70% (-17.58%, -1.45%)
	Week 11	-372.03 (-791.1, 36.74)	-7.59% (-15.45%, 0.8%)	Week 10	-369.10 (-778.91, 29.73)	-7.80% (-15.66%, 0.66%)
ED	Week 0	-88.58 (-184.28, 4.32)	-16.76% (-34.26%, 0.82%)	Week 0	-201.57 (-389.15, -19.49)	-23.56% (-44.47%, -2.38%)
	Week 1	-67.23 (-135.6, 1.82)	-12.67% (-25.42%, 0.36%)	Week 1	-164.2 (-298.21, -28.86)	-19.11% (-34.09%, -3.59%)
	Week 2	-56.03 (-114.83, 0.79)	-10.53% (-20.95%, 0.15%)	Week 2	-144.85 (-260.09, -33.49)	-16.79% (-29.23%, -4.06%)
	Week 4	-44.22 (-91.94, 4.82)	-8.26% (-16.65%, 0.96%)	Week 4	-124.94 (-218.46, -28.82)	-14.36% (-24.21%, -3.57%)
	Week 8	-35.92 (-82.11, 9.63)	-6.63% (-14.75%, 1.87%)	Week 8	-112.29 (-202.84, -23.02)	-12.73% (-22.08%, -2.75%)
Inpatient	Week 0	-54.54 (-119.38 ,8.39)	-24.01% (-51.22% ,3.88%)	Week 0	-98.69 (-198.72 ,-1.6)	-39.68% (-77.03% ,-0.65%)
	Week 1	-30.21 (-76.53 ,16.57)	-13.17% (-32.95% ,7.73%)	Week 1	-73.31 (-144.77 ,-1.14)	-29.3% (-56.97% ,-0.52%)
	Week 2	-16.86 (-56.7 ,21.63)	-7.23% (-23.71% ,10.02%)	Week 2	-59.18 (-120.63 ,0.2)	-23.51% (-45.55% ,0.09%)
	Week 4	-1.64 (-33.96 ,31.59)	-0.46% (-13.95% ,15.31%)	Week 4	-42.65 (-92.52 ,8.61)	-16.72% (-34.61% ,3.93%)
	Week 8	12.23 (-19.07 ,43.09)	5.69% (-8% ,20.76%)	Week 8	-26.54 (-74.83 ,21.06)	-10.09% (-27.22% ,9.27%)

© 2021 Carrel M et al. *JAMA Network Open.*

eTable 2. Estimates and *P* Values for ITS Models for Healthcare Utilization Stratified by Flood Status With Interaction Terms for Race.

**PCP Flooded by Race**					**PCP Non-Flooded by Race**				
**Variable**	**Estimate**	**StdErr**	**tValue**	**Probt**	**Variable**	**Estimate**	**StdErr**	**tValue**	**Probt**
Intercept	3942	115.4825	34.13	<.0001	Intercept	3832	119.2313	32.14	<.0001
series_Week	8.67	1.8629	4.65	<.0001	series_Week	9.0714	1.9792	4.58	<.0001
phase	-2429	313.3391	-7.75	<.0001	phase	-2200	340.8399	-6.46	<.0001
post_week	-50.0255	6.5596	-7.63	<.0001	post_week	-48.6801	7.0815	-6.87	<.0001
log_post_week	1017	143.1585	7.11	<.0001	log_post_week	946.0021	155.1864	6.1	<.0001
WHITE	111.0524	153.2642	0.72	0.4693	WHITE	252.8256	160.4367	1.58	0.1161
series_Week*WHITE	-0.522	2.6586	-0.2	0.8445	series_Week*WHITE	-1.1656	2.8221	-0.41	0.6799
phase*WHITE	13.9809	451.8338	0.03	0.9753	phase*WHITE	-96.0147	492.7604	-0.19	0.8456
post_week*WHITE	-6.2822	9.8417	-0.64	0.5238	post_week*WHITE	-4.1412	10.6424	-0.39	0.6975
log_post_week*WHITE	58.8478	208.6111	0.28	0.7781	log_post_week*WHITE	53.2148	226.6216	0.23	0.8145
AR5	-0.0809	0.0513	-1.58	0.1161	AR5	-0.0878	0.0499	-1.76	0.0795
AR15	0.1499	0.0544	2.76	0.0062	AR13	0.0987	0.0531	1.86	0.064
AR51	-0.2344	0.0552	-4.25	<.0001	AR15	0.1225	0.053	2.31	0.0215
AR52	-0.3972	0.0559	-7.11	<.0001	AR51	-0.2243	0.055	-4.08	<.0001
					AR52	-0.4038	0.0556	-7.26	<.0001
**ED Flooded by Race**					**ED Non-Flooded by Race**				
**Variable**	**Estimate**	**StdErr**	**tValue**	**Probt**	**Variable**	**Estimate**	**StdErr**	**tValue**	**Probt**
Intercept	950.5038	18.8621	50.39	<.0001	Intercept	616.1792	20.661	29.82	<.0001
series_Week	3.9492	0.3595	10.99	<.0001	series_Week	2.9056	0.4093	7.1	<.0001
phase	-274.0152	61.5771	-4.45	<.0001	phase	-239.4493	67.7007	-3.54	0.0005
post_week	-7.2113	1.2792	-5.64	<.0001	post_week	-6.2373	1.41	-4.42	<.0001
log_post_week	57.998	28.1566	2.06	0.0403	log_post_week	67.5123	31.0671	2.17	0.0306
WHITE	-386.0678	26.5705	-14.53	<.0001	WHITE	-158.7734	28.991	-5.48	<.0001
series_Week*WHITE	-1.5357	0.509	-3.02	0.0028	series_Week*WHITE	-2.1628	0.5753	-3.76	0.0002

© 2021 Carrel M et al. *JAMA Network Open.*

phase*WHITE	96.8603	87.3732	1.11	0.2685	phase*WHITE	177.6329	96.2736	1.85	0.066
post_week*WHITE	2.4984	1.8337	1.36	0.1741	post_week*WHITE	3.4821	2.0325	1.71	0.0877
log_post_week*WHITE	-17.6811	39.8791	-0.44	0.6578	log_post_week*WHITE	-41.1751	44.1851	-0.93	0.3521
AR7	0.1431	0.0538	2.66	0.0083	AR24	0.165	0.0564	2.93	0.0037
AR46	0.0987	0.0547	1.8	0.0721	AR25	0.1311	0.0563	2.33	0.0205
AR52	-0.3458	0.0551	-6.28	<.0001	AR44	-0.2066	0.0572	-3.61	0.0004
**Admissions Flooded by Race**					**Admissions Non-Flooded by Race**				
**Variable**	**Estimate**	**StdErr**	**tValue**	**Probt**	**Variable**	**Estimate**	**StdErr**	**tValue**	**Probt**
Intercept	271.2084	10.6273	25.52	<.0001	Intercept	211.1268	13.2606	15.92	<.0001
series_Week	1.0752	0.1916	5.61	<.0001	series_Week	0.3722	0.2758	1.35	0.1781
phase	-88.3427	32.2301	-2.74	0.0065	phase	-107.4093	40.6032	-2.65	0.0086
post_week	-2.6925	0.6707	-4.01	<.0001	post_week	-2.149	0.891	-2.41	0.0165
log_post_week	43.6546	14.743	2.96	0.0033	log_post_week	48.4961	19.3984	2.5	0.0129
WHITE	-46.754	14.8053	-3.16	0.0018	WHITE	-4.5291	18.7469	-0.24	0.8093
series_Week*WHITE	-0.1686	0.2727	-0.62	0.537	series_Week*WHITE	-0.1717	0.3899	-0.44	0.66
phase*WHITE	7.7866	45.9743	0.17	0.8656	phase*WHITE	59.0416	57.1802	1.03	0.3026
post_week*WHITE	-0.4132	0.9777	-0.42	0.6729	post_week*WHITE	0.0106	1.2675	0.01	0.9933
log_post_week*WHITE	-2.2553	21.0296	-0.11	0.9147	log_post_week*WHITE	-11.4757	27.443	-0.42	0.6761
AR1	-0.1343	0.0534	-2.51	0.0125	AR29	0.1644	0.0592	2.78	0.0058
AR19	0.1251	0.0539	2.32	0.0209	AR49	0.1416	0.0596	2.38	0.018
AR52	-0.3891	0.0558	-6.98	<.0001					

© 2021 Carrel M et al. *JAMA Network Open.*

eTable 3. Absolute and Relative Effects of Hurricane Harvey on PCP Visits, ED Visits and Inpatient Admissions in Priority Category 5 and Non–Priority Category 5 Veterans With Flooded and Nonflooded Residential Status at Weeks 0, 1, 2, 4, 8 and, if Applicable, the Week That Visits Were No Longer Significantly Different Than Expected.

		**Flooded**				
		**Priority Category 5**			**Non-Priority Category 5**	
		**Absolute Decline (95% CI)**	**Relative Decline (95% CI)**		**Absolute Decline (95% CI)**	**Relative Decline (95% CI)**
PCP	Week 0	-2805 (-3608.48, -2041.76)	-56.4% (-71.17%, -41.79%)	Week 0	-2223.54 (-2979.52, -1505.41)	-47.88% (-62.86%, -32.98%)
	Week 1	-2042.66 (-2609.36, -1471.81)	-40.95% (-51.83%, -30.08%)	Week 1	-1590.52 (-2123.72, -1052.98)	-34.18% (-45.11%, -23.21%)
	Week 2	-1623.44 (-2109.44, -1148.33)	-32.44% (-41.09%, -23.8%)	Week 2	-1240.75 (-1698.02, -793.73)	-26.61% (-35.54%, -17.72%)
	Week 4	-1142.93 (-1540.83, -735.06)	-22.7% (-29.72%, -15.27%)	Week 4	-836.71 (-1211.08, -452.94)	-17.86% (-25.14%, -10.16%)
	Week 8	-699.39 (-1087.08, -320.37)	-13.72% (-20.51%, -6.68%)	Week 8	-455.81 (-820.58, -99.19)	-9.63% (-16.74%, -2.26%)
				Week 10	-357.15 (-728.84, 4.59)	-7.5% (-14.65%, 0.1%)
ED	Week 0	-362.6 (-536.26 ,-194.02)	-28.19% (-41.06%, -15.53%)	Week 0	-163.93 (-274.09 ,-56.99)	-18.15% (-30.03%, -6.51%)
	Week 1	-315.76 (-439.83 ,-190.46)	-24.44% (-33.62%, -15.03%)	Week 1	-145.09 (-223.79 ,-65.61)	-16.02% (-24.57%, -7.47%)
	Week 2	-292.58 (-399.28 ,-189.48)	-22.55% (-30.14%, -15%)	Week 2	-135.8 (-203.48 ,-70.4)	-14.95% (-21.9%, -7.95%)
	Week 4	-270.9 (-357.49 ,-181.9)	-20.7% (-26.66%, -14.4%)	Week 4	-127.18 (-182.1 ,-70.72)	-13.93% (-19.48%, -8.02%)
	Week 8	-263.22 (-347.05 ,-180.56)	-19.79% (-25.33%, -14.2%)	Week 8	-124.33 (-177.5 ,-71.9)	-13.48% (-18.75%, -8.13%)
Inpatient	Week 0	-148.6 (-247.52, -52.58)	-31.49% (-51.32%, -11.7%)	Week 0	-55.18 (-102.68, -9.06)	-18.88% (-34.56%, -3.22%)
	Week 1	-104.25 (-174.92, -32.88)	-21.97% (-36.21%, -7.33%)	Week 1	-37.96 (-71.9, -3.68)	-12.93% (-24.38%, -1.32%)
	Week 2	-80.46 (-141.23, -21.73)	-16.85% (-28.69%, -4.75%)	Week 2	-28.73 (-57.91, -0.52)	-9.74% (-19.17%, -0.18%)
	Week 4	-54.32 (-103.64, -3.63)	-11.24% (-20.69%, -0.81%)	Week 3	-22.76 (-47.96, 2.57)	-7.68% (-15.9%, 0.9%)
	Week 5	-46.48 (-94.72, 2.41)	-9.55% (-18.67%, 0.55%)	Week 4	-18.59 (-42.27, 5.76)	-6.24% (-13.79%, 2.06%)
	Week 8	-33.05 (-80.8, 14.03)	-6.66% (-15.84%, 3.08%)	Week 8	-10.35 (-33.28, 12.26)	-3.4% (-10.83%, 4.3%)
		**Non-Flooded**				
		**Priority Category 5**			**Non-Priority Category 5**	
		**Absolute Decline (95% CI)**	**Relative Decline (95% CI)**		**Absolute Decline (95% CI)**	**Relative Decline (95% CI)**
PCP	Week 0	-2367.82 (-3276.5, -1504.65)	-44.39% (-60.21%, -28.73%)	Week 0	-2102.61 (-2941.05, -1306.16)	-45.95% (-62.82%, -29.11%)
	Week 1	-1794 (-2434.9, -1147.89)	-33.5% (-44.93%, -22.04%)	Week 1	-1495.17 (-2086.52, -899.01)	-32.61% (-44.92%, -20.22%)
	Week 2	-1485.02 (-2034.65, -947.71)	-27.63% (-36.96%, -18.37%)	Week 2	-1159.03 (-1666.17, -663.23)	-25.22% (-35.29%, -15.15%)
	Week 4	-1143.35 (-1593.34, -682.06)	-21.11% (-28.59%, -13.21%)	Week 4	-769.77 (-1184.98, -344.13)	-16.67% (-24.89%, -7.9%)

© 2021 Carrel M et al. *JAMA Network Open.*

	Week 8	-859.46 (-1297.91, -430.82)	-15.64% (-22.66%, -8.34%)	Week 8	-400.43 (-804.99, -4.92)	-8.57% (-16.62%, -0.11%)
				Week 9	-347.31 (-752.41, 52.06)	-7.41% (-15.4%, 1.19%)
ED	Week 0	-134.62 (-329.08 ,54.13)	-16.39% (-39.24%, 6.98%)	Week 0	-127.6 (-226.35 ,-31.75)	-22.75% (-39.61%, -5.92%)
	Week 1	-121.44 (-260.36 ,18.87)	-14.71% (-31.21%, 2.42%)	Week 1	-96.79 (-167.34 ,-25.54)	-17.21% (-29.49%, -4.75%)
	Week 2	-116.61 (-236.07 ,-1.16)	-14.06% (-27.55%, -0.15%)	Week 2	-80.11 (-140.78 ,-21.48)	-14.2% (-24.23%, -3.94%)
	Week 4	-115.67 (-212.62 ,-16.02)	-13.82% (-24.46%, -2.08%)	Week 4	-61.48 (-110.71 ,-10.87)	-10.84% (-18.98%, -2.04%)
	Week 8	-126.4 (-220.26 ,-33.85)	-14.87% (-24.78%, -4.31%)	Week 8	-45.52 (-93.19 ,1.48)	-7.94% (-15.79%, 0.27%)
Inpatient	Week 0	-129.1 (-261.08, -0.99)	-35.56% (-69.53%, -0.27%)	Week 0	-41.66 (-96.2, 11.29)	-20.83% (-46.89%, 6%)
	Week 1	-100.41 (-194.7, -5.18)	-27.47% (-52.48%, -1.59%)	Week 1	-19.68 (-58.65, 19.67)	-9.72% (-28.98%, 10.35%)
	Week 2	-84.91 (-165.99, -6.55)	-23.08% (-43%, -1.92%)	Week 2	-7.5 (-41.01, 24.88)	-3.56% (-19.59%, 13.21%)
	Week 4	-67.66 (-133.46, -0.03)	-18.17% (-34.23%, -0.01%)	Week 4	6.65 (-20.54, 34.61)	3.61% (-9.66%, 19.05%)
	Week 5	-62.39 (-126.76, 2.83)	-16.66% (-31.45%, 0.92%)	Week 5	11.24 (-15.36, 38.2)	5.94% (-7.15%, 21.02%)
	Week 8	-53.07 (-116.78, 9.74)	-13.93% (-28.95%, 2.94%)	Week 8	20.2 (-6.13, 46.16)	10.48% (-2.96%, 25.39%)

© 2021 Carrel M et al. *JAMA Network Open.*

eTable 4. Estimates and *P* Values for ITS Models for Healthcare Utilization Stratified by Flood Status With Interaction Terms for Priority Category (Income).

**PCP Flooded by Income**					**PCP Non-Flooded by Income**				
**Variable**	**Estimate**	**StdErr**	**tValue**	**Probt**	**Variable**	**Estimate**	**StdErr**	**tValue**	**Probt**
Intercept	4050	141.7697	28.56	<.0001	Intercept	4088	134.2173	30.46	<.0001
series_Week	6.8953	2.0865	3.3	0.0011	series_Week	5.8251	2.0922	2.78	0.0057
phase	-2260	329.2677	-6.86	<.0001	phase	-2134	351.0656	-6.08	<.0001
post_week	-49.6676	7.0629	-7.03	<.0001	post_week	-44.0939	7.3535	-6	<.0001
log_post_week	997.9621	152.1546	6.56	<.0001	log_post_week	924.3372	160.2954	5.77	<.0001
priority_5	-130.355	178.829	-0.73	0.4666	priority_5	-248.096	175.2884	-1.42	0.158
series_Week*priority_5	5.3391	2.9905	1.79	0.0752	series_Week*priority_5	11.6413	2.9968	3.88	0.0001
phase*priority_5	-598.097	473.2098	-1.26	0.2072	phase*priority_5	-336.721	507.351	-0.66	0.5074
post_week*priority_5	-15.6643	10.497	-1.49	0.1367	post_week*priority_5	-21.9806	11.0293	-1.99	0.0472
log_post_week*priority_5	220.3868	220.2154	1	0.3177	log_post_week*priority_5	37.9843	233.9092	0.16	0.8711
AR5	-0.0928	0.0527	-1.76	0.0795	AR2	-0.1056	0.0495	-2.13	0.0337
AR51	-0.2062	0.0565	-3.65	0.0003	AR3	0.1176	0.0501	2.35	0.0197
AR52	-0.4058	0.0565	-7.18	<.0001	AR5	-0.0824	0.0495	-1.66	0.0974
					AR13	0.1146	0.0518	2.21	0.0277
					AR51	-0.2438	0.0538	-4.53	<.0001
					AR52	-0.4119	0.0538	-7.65	<.0001
**ED Flooded by Income**					**ED Non-Flooded by Income**				
**Variable**	**Estimate**	**StdErr**	**tValue**	**Probt**	**Variable**	**Estimate**	**StdErr**	**tValue**	**Probt**
Intercept	705.4893	28.9696	24.35	<.0001	Intercept	484.0956	24.6351	19.65	<.0001
series_Week	2.2376	0.474	4.72	<.0001	series_Week	0.8443	0.4886	1.73	0.085
phase	-145.524	68.4754	-2.13	0.0344	phase	-107.439	69.631	-1.54	0.1239
post_week	-4.2585	1.5064	-2.83	0.005	post_week	-3.3615	1.5227	-2.21	0.028
log_post_week	30.0562	31.9519	0.94	0.3476	log_post_week	45.2057	32.3396	1.4	0.1632
priority_5	118.6353	39.2786	3.02	0.0027	priority_5	47.2889	34.8183	1.36	0.1754

© 2021 Carrel M et al. *JAMA Network Open.*

series_Week*priority_5	3.044	0.6826	4.46	<.0001	series_Week*priority_5	2.4086	0.6911	3.49	0.0006
phase*priority_5	-157.219	97.553	-1.61	0.1081	phase*priority_5	-1.8045	98.6609	-0.02	0.9854
post_week*priority_5	-5.1654	2.173	-2.38	0.0181	post_week*priority_5	-3.0938	2.1457	-1.44	0.1504
log_post_week*priority_5	29.2482	45.3944	0.64	0.5199	log_post_week*priority_5	-27.0344	45.9988	-0.59	0.5572
AR3	-0.1184	0.0559	-2.12	0.0349	AR38	-0.1859	0.0626	-2.97	0.0032
AR18	-0.1355	0.0567	-2.39	0.0173	AR46	0.1492	0.0635	2.35	0.0194
AR52	-0.3766	0.0597	-6.31	<.0001					
**Admissions Flooded by Income**					**Admissions Non-Flooded by Income**				
**Variable**	**Estimate**	**StdErr**	**tValue**	**Probt**	**Variable**	**Estimate**	**StdErr**	**tValue**	**Probt**
Intercept	227.6478	13.1403	17.32	<.0001	Intercept	197.6451	14.9706	13.2	<.0001
series_Week	0.7447	0.237	3.14	0.0018	series_Week	0.007882	0.2697	0.03	0.9767
phase	-59.0093	37.747	-1.56	0.119	phase	-37.1645	44.1351	-0.84	0.4004
post_week	-2.0144	0.8061	-2.5	0.013	post_week	-1.2674	0.9153	-1.38	0.1672
log_post_week	28.6083	17.519	1.63	0.1035	log_post_week	29.4322	20.0067	1.47	0.1423
priority_5	78.5468	18.2319	4.31	<.0001	priority_5	47.7585	20.9929	2.27	0.0236
series_Week*priority_5	1.1544	0.3356	3.44	0.0007	series_Week*priority_5	1.3798	0.3857	3.58	0.0004
phase*priority_5	-106.908	53.9129	-1.98	0.0483	phase*priority_5	-98.7802	62.9618	-1.57	0.1177
post_week*priority_5	-3.7972	1.1832	-3.21	0.0015	post_week*priority_5	-1.8292	1.3258	-1.38	0.1687
log_post_week*priority_5	54.8113	25.1446	2.18	0.03	log_post_week*priority_5	16.0249	28.5928	0.56	0.5756
AR1	-0.148	0.054	-2.74	0.0065	AR9	0.1344	0.0573	2.35	0.0197
AR32	0.1582	0.058	2.72	0.0068	AR12	-0.1134	0.0581	-1.95	0.0519
AR52	-0.4263	0.061	-6.99	<.0001	AR47	-0.2227	0.0641	-3.47	0.0006
					AR52	-0.1517	0.066	-2.3	0.0223

© 2021 Carrel M et al. *JAMA Network Open.*

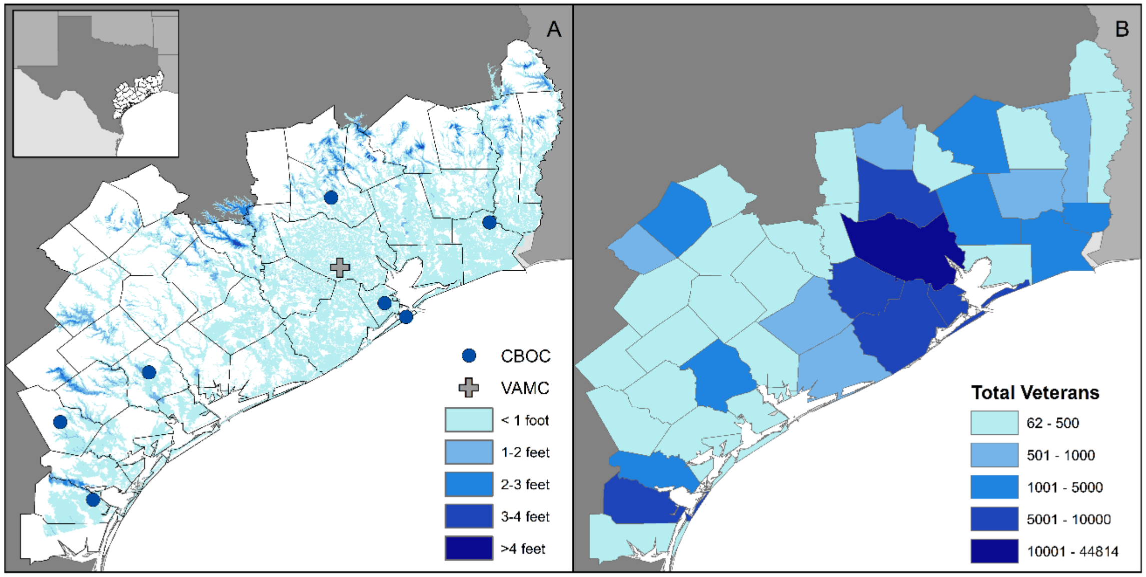

eFigure 1. Flooded Areas in Texas Following Hurricane Harvey With Locations of VHA Care (Houston VA Medical Center [VAMC] and Community Based Outpatient Clinics [CBOCs]) and Total Veterans in Disaster Declared Counties.

© 2021 Carrel M et al. *JAMA Network Open.*

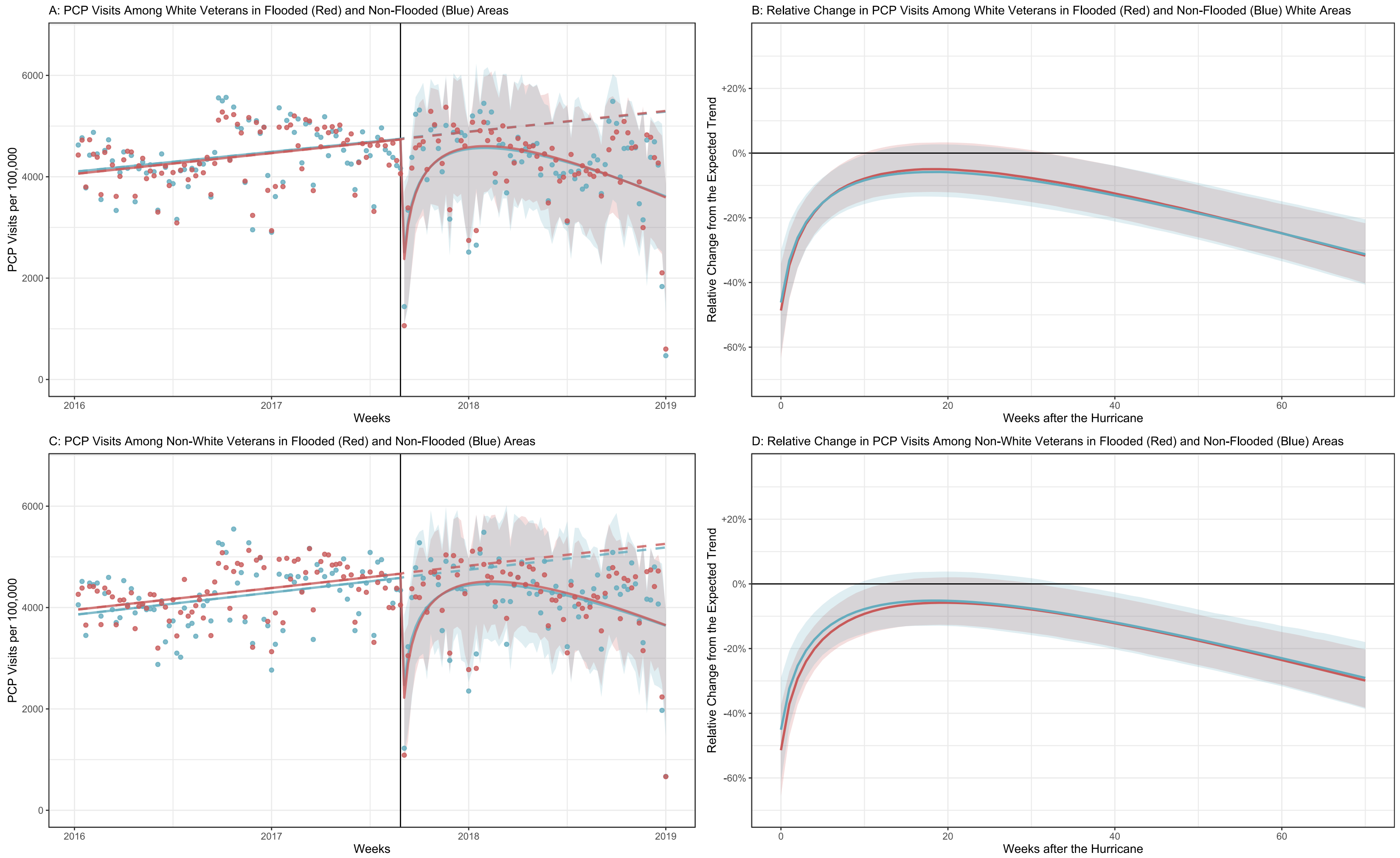

eFigure 2. Absolute and Relative Changes in PCP Visits Among Veterans With Flooded (Red) and Nonflooded (Blue) Residences Who Were White (A, B) and Non-White (C,D). Solid lines fit observed weekly data points, dashed lines indicate trends if no hurricane had occurred, shaded areas represent 95% confidence intervals (A, C). Weekly percent relative change from expected trend are solid lines and shaded areas represent 95% confidence intervals (B, D).

© 2021 Carrel M et al. *JAMA Network Open.*

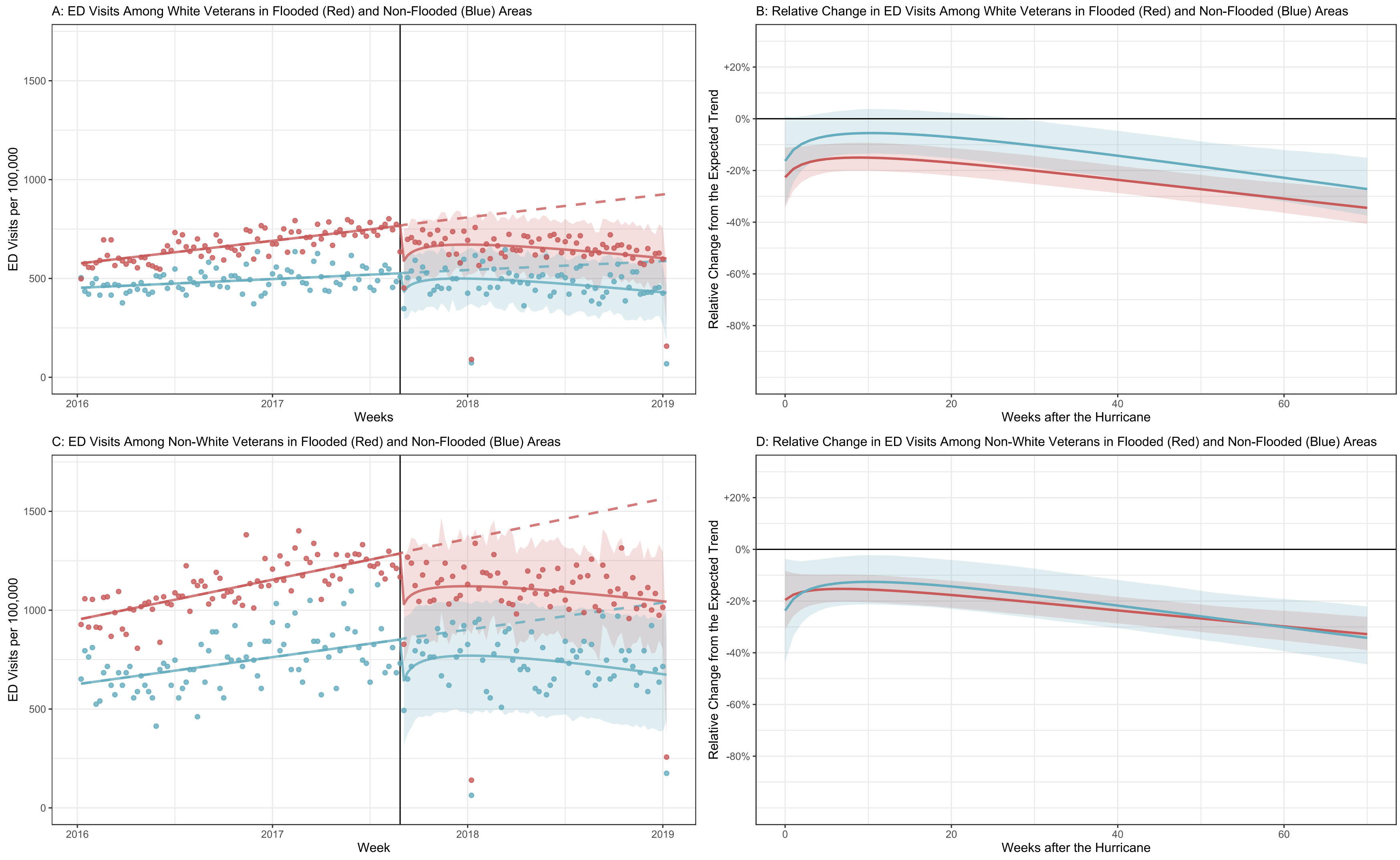

eFigure 3. Absolute and Relative Changes in ED Visits Among Veterans With Flooded (Red) and Nonflooded (Blue) Residences Who Were White (A, B) and Non-White (C,D). Solid lines fit observed weekly data points, dashed lines indicate trends if no hurricane had occurred, shaded areas represent 95% confidence intervals (A, C). Weekly percent relative change from expected trend are solid lines and shaded areas represent 95% confidence intervals (B, D).

© 2021 Carrel M et al. *JAMA Network Open.*

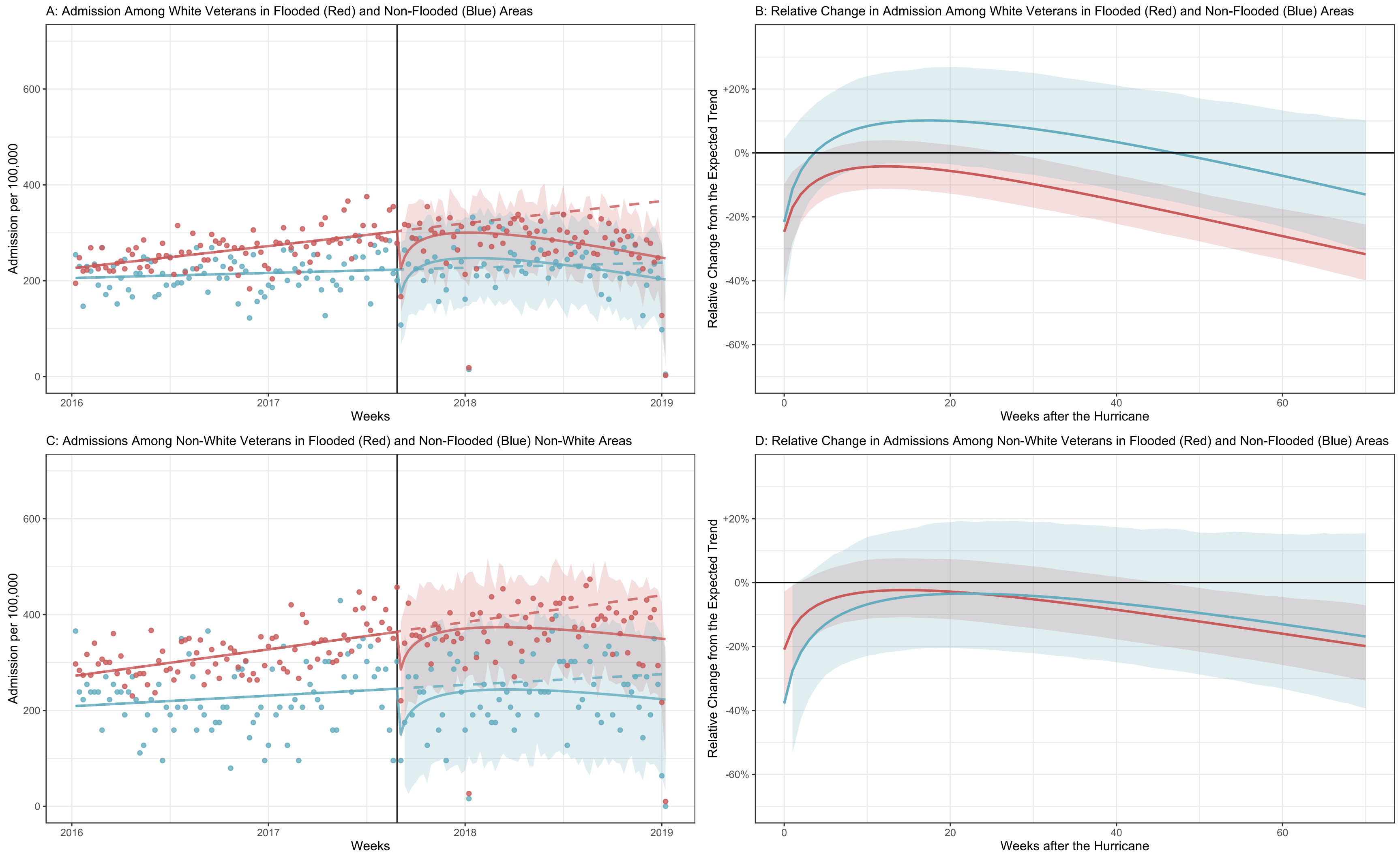

eFigure 4. Absolute and Relative Changes in Inpatient Admissions Among Veterans With Flooded (Red) and Nonflooded (Blue) Residences Who Were White (A, B) and Non-White (C,D). Solid lines fit observed weekly data points, dashed lines indicate trends if no hurricane had occurred, shaded areas represent 95% confidence intervals (A, C). Weekly percent relative change from expected trend are solid lines and shaded areas represent 95% confidence intervals (B, D).

© 2021 Carrel M et al. *JAMA Network Open.*

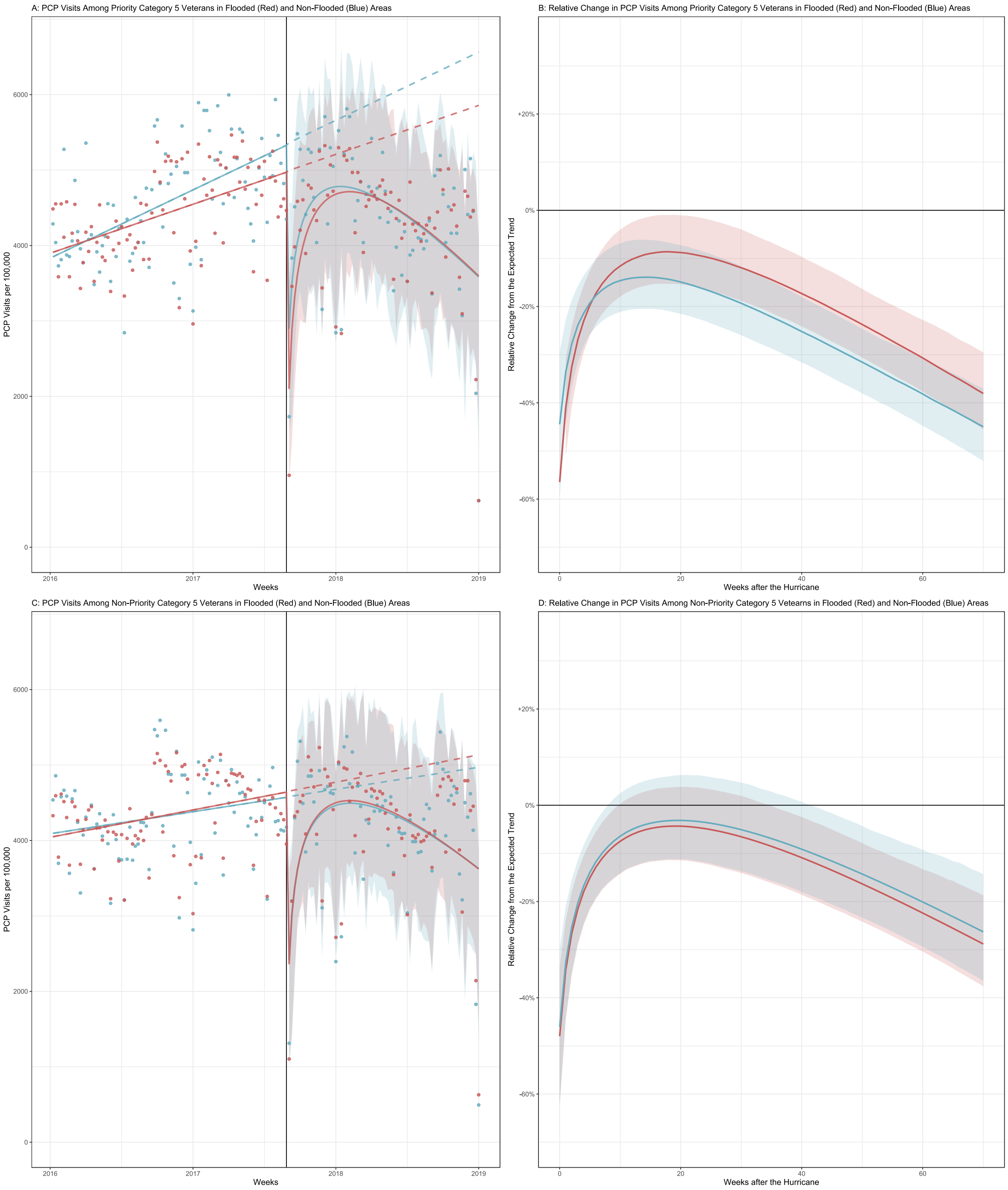

eFigure 5. Absolute and Relative Changes in PCP Visits Among Veterans With Flooded (Red) and Nonflooded (Blue) Residences Who Were Priority Category 5 (A, B) and Non–Priority Category 5 (C,D). Solid lines fit observed weekly data points, dashed lines indicate trends if no hurricane had occurred, shaded areas represent 95% confidence intervals (A, C). Weekly percent relative change from expected trend are solid lines and shaded areas represent 95% confidence intervals (B, D).

.

© 2021 Carrel M et al. *JAMA Network Open.*

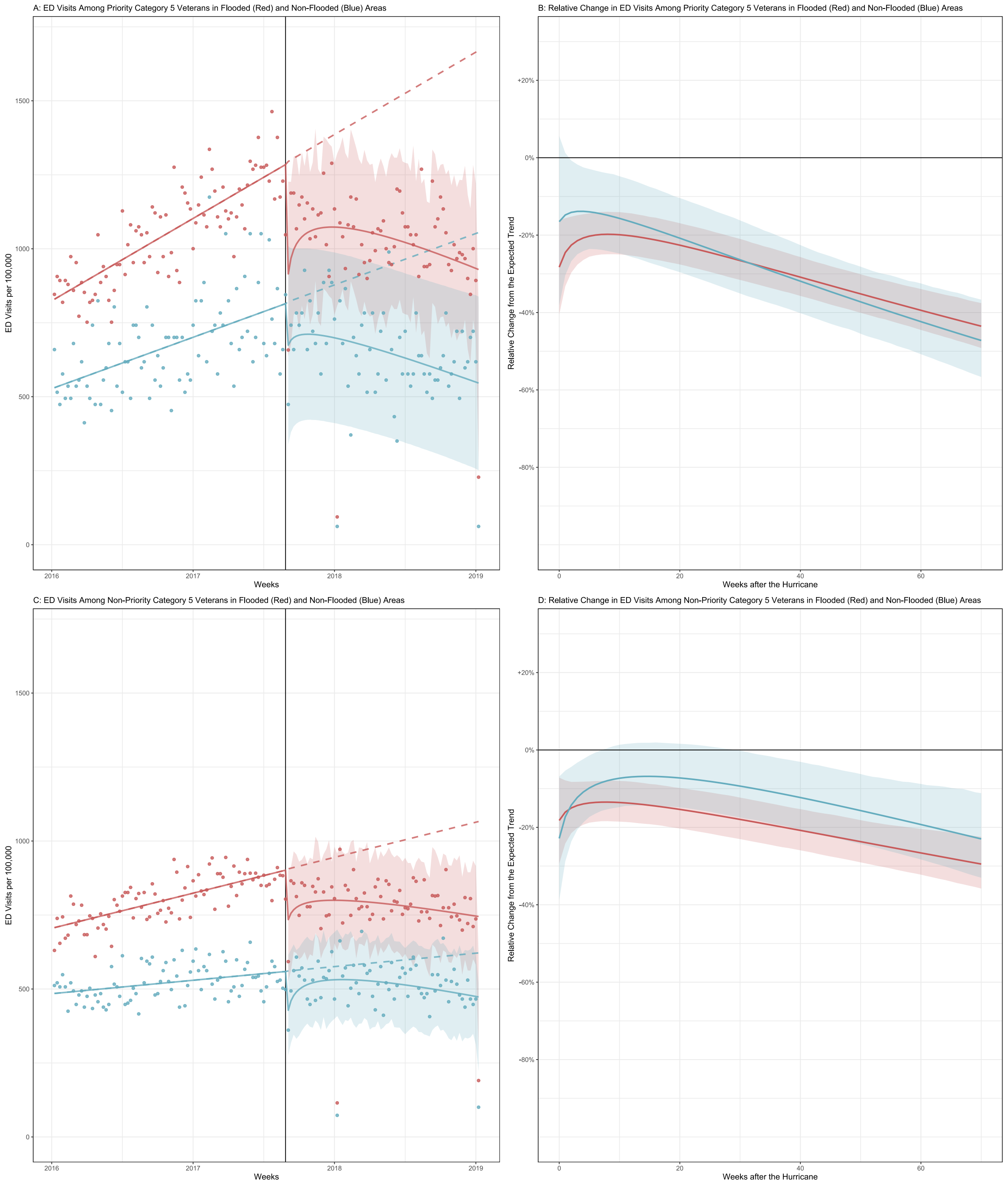

eFigure 6. Absolute and Relative Changes in ED Visits Among Veterans With Flooded (Red) and Nonflooded (Blue) Residences Who Were Priority Category 5 (A, B) and Non–Priority Category 5 (C,D). Solid lines fit observed weekly data points, dashed lines indicate trends if no hurricane had occurred, shaded areas represent 95% confidence intervals (A, C). Weekly percent relative change from expected trend are solid lines and shaded areas represent 95% confidence intervals (B, D).

© 2021 Carrel M et al. *JAMA Network Open.*

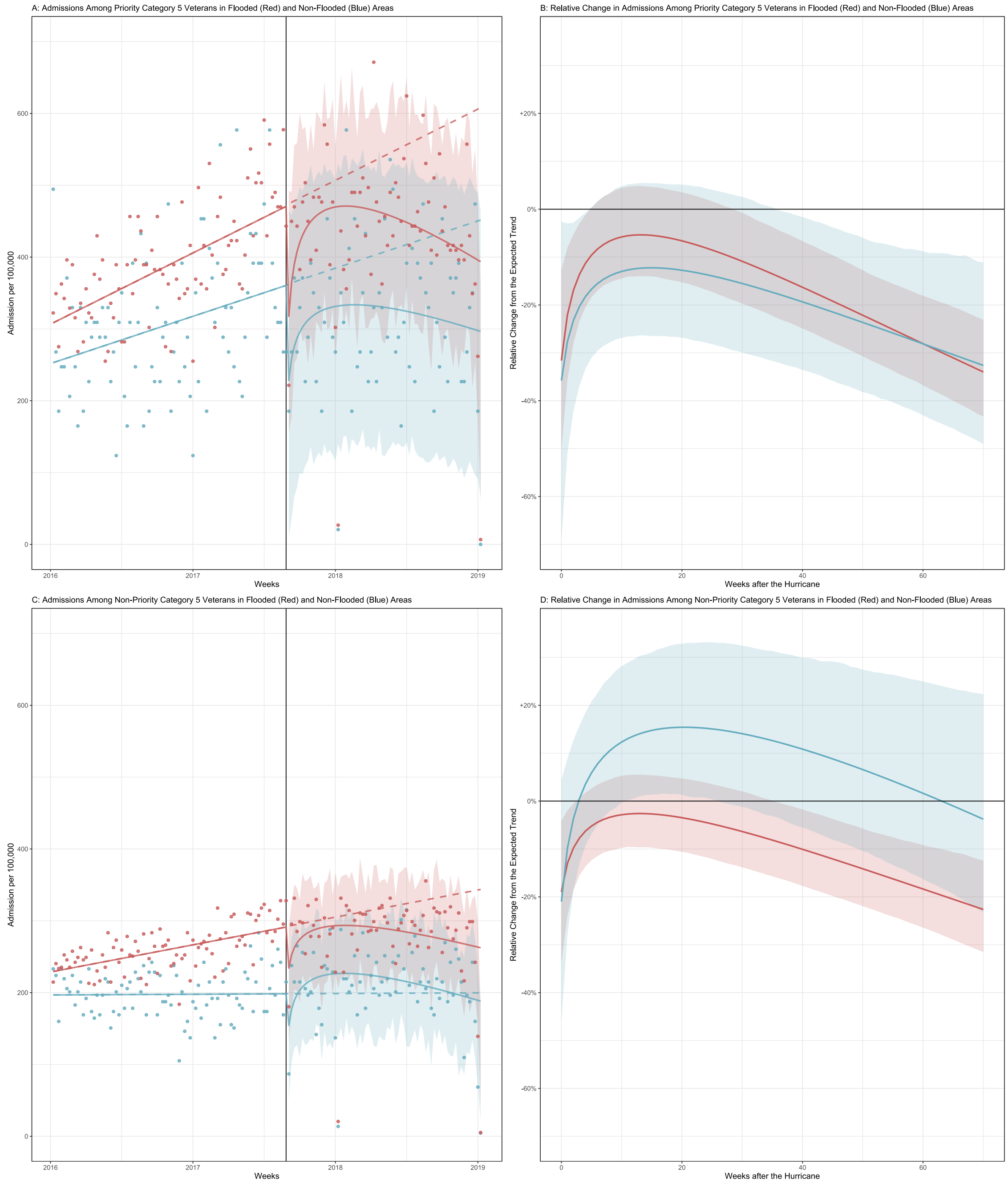

eFigure 7. Absolute and Relative Changes in Inpatient Admissions Among Veterans With Flooded (Red) and Nonflooded (Blue) Residences Who Were Priority Category 5 (A, B) and Non–Priority Category 5 (C,D). Solid lines fit observed weekly data points, dashed lines indicate trends if no hurricane had occurred, shaded areas represent 95% confidence intervals (A, C). Weekly percent relative change from expected trend are solid lines and shaded areas represent 95% confidence intervals (B, D).

© 2021 Carrel M et al. *JAMA Network Open.*

eAppendix. Example SAS Code for ITS Models and Bootstrapping of Confidence Intervals.

For details on the bootstrapping macro, see http://www.dacp.org/pdf/macro_boots_jce.sas.txt, developed by Zhang, et. al. https://doi.org/10.1016/j.jclinepi.2008.08.007

```
/*absolute and relative change in admissions boots method*/

/*setting the variables to match the macro*/
data WKLY_RTS_adm;set pdrive.per_week_adm ;
     week=_n_;
     intervention=(week>=87);
     weekafintervention=max(0,week-86);

      if weekafintervention = 0 then log_post_week =
log(weekafintervention + 1);
     else log_post_week = log(weekafintervention);
run;

/*generating ITS parameter estimates*/

proc autoreg data= WKLY_RTS_adm;
     model fl1_rt100k=week intervention weekafintervention
log_post_week/nlag=(52) method=ml covb maxiter=1000;
      ods output ParameterEstimates=pre_boot1;
run;

*----------------;
*Boots method;
*----------------;

/*FLOODED*/
options nonotes nosyntaxcheck nosymbolgen;
%boots(244.5391,0.9724,-65.0300,-2.2349, 29.6302,
156,        1334,%str(52),1500,86,52,
      time intervention1 timeafterintervention1  log_post_week);

data pdrive.bootsim_adm_fl1;
set simulationresults(firstobs=2);
run;

/*Generating absolute and relative values for specific weeks*/

%macro week(wk);
%macro nm(vr);
data meanvar;
     set pdrive.bootsim_adm_fl1;
      lvrel=intervention1/intercept;
      slrel=timeafterintervention1/time;


abs&wk.=intervention1+&wk.*timeafterintervention1+log(&wk.+1)*log_post
_week;
```

© 2021 Carrel M et al. *JAMA Network Open.*

```
rel&wk.=(intervention1+&wk.*timeafterintervention1+log(&wk.+1)*log_pos
t_week)/(intercept+(86+&wk.)*time);

run;


proc univariate data=meanvar alpha=0.05;
        var &vr;
         output out=&vr pctlpre=col pctlpts=2.5,50,97.5 mean=me
        ;
run;
data &vr;
set &vr;
wk=&wk.;
run;
proc print;run;
%mend nm;
%nm(rel&wk.);
%nm(abs&wk.); %mend week;
%week(0);
%week(1);
%week(2);
%week(3);
%week(4);
%week(5);
%week(6);
%week(7);
%week(8);
%week(9);
%week(10);
%week(11);
%week(12);
%week(13);
%week(14);
%week(15);
%week(16);
%week(17);
%week(18);
%week(19);
%week(20);


data pdrive.abs_adm_fl1;
set abs0-abs20;
run;
proc print data=pdrive.abs_adm_fl1; run;
data pdrive.rel_adm_fl1;
set rel0-rel20;
run;
```

© 2021 Carrel M et al. *JAMA Network Open.*
